# ISL1 promoted tumorigenesis and EMT via Aurora kinase A-induced activation of PI3K/AKT signaling pathway in neuroblastoma

**DOI:** 10.1038/s41419-021-03894-3

**Published:** 2021-06-15

**Authors:** Mengzhen Li, Chengtao Sun, Xiaoyun Bu, Yi Que, Lian Zhang, Yu Zhang, Li Zhang, Suying Lu, Junting Huang, Jia Zhu, Juan Wang, Feifei Sun, Yizhuo Zhang

**Affiliations:** 1 Sun Yat-sen University Cancer Center; State Key Laboratory of Oncology in South China; Collaborative Innovation Center for Cancer Medicine, Guangzhou, Guangdong 510060 P. R. China; 2 Department of Pediatric Oncology, Sun Yat-sen University Cancer Center, Guangzhou, Guangdong 510060 P. R. China; 3 Department of Oncology, The First Affiliated Hospital of Shandong First Medical University & Shandong Provincial Qianfoshan Hospital, Shandong Lung Cancer Institute, Jinan, Shandong 250014 P. R. China; 4 Department of Hepatobiliary Oncology, Sun Yat-sen University Cancer Center, State Key Laboratory of Oncology in South China, Collaborative Innovation Center for Cancer Medicine, Guangzhou, China; 5 Fifth Affiliated Hospital, Guangzhou Medical University, Minimally Invasive Technique and Product Translational Center, Guangzhou Medical University, Guangzhou, 510700 P. R. China

**Keywords:** Metastasis, Paediatric cancer, Oncogenesis

## Abstract

Neuroblastoma (NB) is the most common extracranial solid malignancy in children and its mortality rate is relatively high. However, driver genes of NB are not clearly identified. Using bioinformatics analysis, we determined the top 8 differentially expressed genes (DEGs) in NB, including GFAP, PAX6, FOXG1, GAD1, PTPRC, ISL1, GRM5, and GATA3. Insulin gene enhancer binding protein 1 (ISL1) is a LIM homeodomain transcription factor which has been found to be highly expressed in a variety of malignant tumors, but the function of ISL1 in NB has not been fully elucidated. We identified ISL1 as an oncogene in NB. ISL1 is preferentially upregulated in NB tissues compared with normal tissues. High ISL1 expression is significantly associated with poor outcome of NB patients. Knockdown of ISL1 markedly represses proliferation and induces cell apoptosis in vitro, and suppresses tumorigenicity in vivo, while overexpression of ISL1 has the opposite effects. Mechanistically, we demonstrate that ISL1 promotes cell proliferation and EMT transformation through PI3K/AKT signaling pathway by upregulating Aurora kinase A (AURKA), a serine-threonine kinase that is essential for the survival of NB cells. The blockade of AURKA attenuates the function of ISL1 overexpression in the regulation of cell proliferation and migration, Conclusively, this study showed that ISL1 targeted AURKA to facilitate the development of NB, which provided new insights into the tumorigenesis of NB. Thus, ISL1 may be a promising therapeutic target in the future.

## Introduction

Neuroblastoma (NB) is the most common extracranial solid malignancy in children and accounts for 12% of pediatric cancer deaths [1]. Although children younger than 1 year had 5-year survival for about 90%, long-term outcomes for children with high-risk NB are still poor [1–3]. Multimodality treatment for patients with high-risk NB includes chemotherapy, surgery, external beam radiation, and immunotherapy [4]. Although anti-GD2-specific mAb immunotherapy has shown significant improvement in the outcome for high-risk NB patients [5, 6], the understanding of the molecular and genetic properties related to NB is still necessary for designing promising treatments for NB patients.

The Gene Expression Omnibus (GEO) database has evolved rapidly in recent years and was gradually used for the bioinformatics mining of gene expression profiles in cancers [7, 8]. In our study, we performed a series of interactive analyses on the GEO datasets, and then extracted the top 8 hub genes. To further explore the relationship between the selected hub genes and the pathogenesis of NB, ISL1 was identified as the key research element for further study.

ISL1, a LIM homeodomain transcription factor, was initially identified as a protein that binds to an insulin gene enhancer and regulates its expression [9]. Earlier it was supposed to be restrictively expressed on neuroendocrine tumors of pancreatic origin. However, recent studies revealed that it could be expressed in many neuroendocrine tumors of extra-pancreatic origin, such as pulmonary small-cell neuroendocrine carcinoma, Merkel cell carcinomas, paragangliomas/pheochromocytomas, medullary thyroid carcinomas, and adrenal neuroblastomas [10]. Recently, ISL1 has been reported to play crucial roles in cancer progression, and this is mainly based on an aberrant expression of ISL1. ISL1 was involved in triple-negative breast cancer, melanoma, and gastric cancer progression [11–13], and it was highly expressed in non-Hodgkin lymphoma compared to normal lymph nodes or Hodgkin lymphoma [14]. Besides, ISL1 was also verified to be a novel regulator of the cyclin D1 and c-Myc genes in cancer [15], and it could predict prognostics for cancers like gastric cancer, bladder cancer, and may also act as a biomarker in NB [13, 16–18]. Nevertheless, the function and underlying mechanisms of ISL1 in NB have not been fully elucidated till now.

As a member of the Aurora kinase family, Aurora kinase A (AURKA) has multiple functions in mitosis and non-mitotic biological processes [19, 20], overexpression of AURKA is implicated in genetic instability and tumorigenesis in various cancers, including ovarian cancer, leukemia, liver cancer, lung cancer, and pancreas cancer [21–27]. It is involved in tumor progression via PI3K/AKT pathway, and also regulates EMT in several cancers [28–31]. High expression of AURKA is associated with poor prognosis in NB [32]. In the present study, we demonstrated that ISL1 promoted tumorigenesis via upregulating AURKA and revealed the related regulatory mechanism. Our study verified the involvement of ISL1 in tumorigenesis and cancer progression of NB, which may help to provide a potential target for NB therapy in the future.

## Materials and methods

### Gene expression profile datasets

Two mRNA expression profiles (GSE3960 and GSE54720) were obtained from NCBI-GEO (https://www.ncbi.nlm.nih.gov/geo/). The microarray data of GSE3960, which included 101 NB primary tumors and 1 fetal brain tissue was based on GPL8300 (Affymetrix Human Genome U95 Version 2 Array, Affymetrix Inc., Santa Clara, CA, USA); while microarray data of GSE54720 was based on the GPL13667 platform (Affymetrix Human Genome U219 Array, Affymetrix Inc., Santa Clara, CA, USA), and included 19 NB tumors and 4 non-pathological tissues. The combined cohort of TCGA, TARGET, and GTEx samples were obtained from UCSC Xena browser (TCGA TARGET GTEx cohort (https://xenabrowser.net/datapages/?cohort=TCGA%20TARGET%20GTEx&removeHub=https%3A%2F%2Fxena.treehouse.gi.ucsc.edu%3A443)), and there were 162 NB samples and 1280 normal tissues (128 normal adrenal gland and 1152 normal brain tissues).

### Differentially expressed gene (DEG) identification

In the present study, the R package *limma* was used for screening differentially expressed genes (DEGs) between NB samples and non-cancerous tissues in GSE3960 and GSE54720. Since the R package *limma* corrected the multiple testing by calculating adjusted *P*-value, we considered adjusted *P* < 0.05 and |log2FC| > 1 as the DEGs cut-off criteria. The heatmap and clustering was done by using R package pheatmap. The Venn diagram and volcano plot were conducted by Echart (http://www.ehbio.com/ImageGP/index.php/Home/Index/index.html).

### Gene Ontology (GO) and protein–protein interaction (PPI) network analysis

GO classification of the terms associated with overlapping DEGs was performed using the online tools of Database for Annotation, Visualization, and Integrated Discovery (DAVID). *P* < 0.05 was defined as the cut-off value for significant function analysis.

The Search Tool for the Retrieval of Interacting Genes (STRING) database was used to analyze the PPI network of overlapping DEGs at the protein level. Additionally, Cytoscape software was employed for visualizing the PPI networks of common DEGs. The hub genes in PPI were also identified using the CytoHubba plugin.

### Prognostic signature generation and prediction

The association between the expression level of identified hub genes and EFS/OS in NB patients was assessed by using RNA-Seq and clinical data from the TARGET-NBL cohort (https://xenabrowser.net). Survival analysis was performed through GraphPad Prism 7.0 (GraphPad Software Inc., USA), and *P*-values < 0.05 were considered statistically significant. Besides, the R2 web-based application (http://r2.amc.nl) was used to generate Kaplan-Meier survival curves of identified hub genes from data in GSE62564 and E-TABM-38.

The ROC curve was used to assess the identified hub genes’ diagnostic efficiency between NB and normal tissues based on the GSE54720 dataset. ROC analysis was performed by SPSS 20 (SPSS Inc., Chicago, IL, USA).

### Clinical samples and cell lines

All procedures of this study were approved by the Ethics Committee of Sun Yat-sen University Cancer Center. Tissue samples and available clinical–pathological data were obtained from the Department of Pediatric Oncology, Sun Yat-sen University Cancer Center (Guangzhou, Guangdong, China) between April 2009 and June 2016, with written informed consent from participants. All cell lines, including HEK293T, SK-N-SH, SK-N-BE (2), SH-SY5Y, IMR32, and SK-N-AS cell lines were purchased from the COBIOER BIOSCIENCES (Nanjing, China). SK-N-SH and IMR32 cell lines were cultured in minimum essential medium (MEM: Gibco, USA). The SK-N-BE(2) and SH-SY5Y cell lines were cultured in MEM/F12 (1:1; MEM: Gibco, USA; F-12 basic: Gibco, USA). HEK293T and SK-N-AS cell lines were cultured in Dulbecco’s modified Eagle’s medium (DMEM: Gibco, USA). Mediums of SK-N-SH, SK-N-BE (2), SH-SY5Y, and IMR32 cell lines were supplemented with 10% FBS (HyClone, USA), 1% penicillin–streptomycin solution (HyClone, USA), 1% 1 mM Sodium Pyruvate (NAP: Gibco, USA) and 1% MEM non-essential amino acids (MEM NEAA: Gibco, USA). The medium of SK-N-AS cell line was supplemented with 10% FBS (HyClone, USA), 1% penicillin–streptomycin solution (HyClone, USA), 1% non-essential amino acids (MEM NEAA: Gibco, USA). All cells were cultured in a 5% CO_2_ and humidified incubator was maintained at 37 °C.

### siRNA transfection

Human siRNA oligos were purchased from GenePharma (Suzhou, China). For transfection, NB cells were plated onto 6-well plates using Lipofectamine 3000 (Invitrogen, USA) according to the manufacturer’s instructions. The transfected cells were cultured for 24 h, then the RNA was harvested and analyzed by quantitative real-time PCR (qRT-PCR) technique. The transfected cells would be cultured for up to 48 h for other functional studies. To maintain the stability of siRNA, we used the 2ʹ-O-me-modified si-ISL1 when injecting into the mice following the manufacturer’s instructions.

### Lentiviral transduction of NB cells

The ISL1 plasmids and lentiviral vectors were purchased from GeneCopoeia (Guangzhou, China). The lentiviral supernatants were produced by transfecting HEK293T cells and concentrated by ultracentrifuging. The concentrated ISL1 lentivirus was immediately stored at −80 °C for further use. SK-N-SH and SK-N-BE (2) cell lines were transduced with lentiviral supernatants in 6-well dishes and 2 μg/mL puromycin was used to acquire the cells 72 h later. The ISL1 expression levels were then examined by western blot (WB) analysis.

### qRT-PCR analysis

We used Trizol reagent (Life Technologies, USA) to extract total RNA from the cultured cells. Prime Script RT Master Mix (TaKaRa, Japan) was used to reverse transcribe the RNA into cDNA following the manufacturer’s instructions. Then qRT-PCR was conducted on the LightCycler 480 (Roche, Basel, Switzerland) system with a Power SYBR Green Master Mix (Dongsheng Biotech, China). β-Actin was used as an endogenous control. Specific primers for 8 hub genes are listed in Supplementary Table 3. All these primers were obtained from (Sangon Biotech, China). Levels of gene expression were calculated by the ΔΔCt method.

### WB analysis and antibodies

The whole-cell lysates were generated using the Whole Cell Lysis Assay Kit (KeyGEN, China) according to the manufacturer’s protocol. The protein concentration was detected by the BCA method (Thermo Fisher Scientific, USA). β-Actin (1: 1000, Abcam, UK) was used as an internal control. Primary antibodies included ISL1 (0.5 μg/mL, Developmental Studies Hybridoma Bank, USA), GATA3 (1:1000, Invitrogen, USA), E-cadherin (1:10,000; Abcam, UK), N-cadherin (1:5000; Abcam, UK), vimentin (1:1000; Abcam, UK), snail + Slug (1 μg/mL; Abcam, UK), Bcl2 (1:1000; Abcam, UK), Bax (1:1000; Abcam, UK), Activated Caspase3 (1:1000; Abcam, UK), AKT (1:1000, Cell Signaling Technology, USA), p-AKT (1:1000, Cell Signaling Technology, USA), and AURKA (1:1000; Abcam, UK). Secondary antibody included goat anti-rabbit antibody (1:10,000, Jacson ImmunoResearch, USA) or goat anti-mouse antibody (1:10,000, Jacson ImmunoResearch, USA). The ECL chemiluminescence detection system (Bio-Rad, USA) was used for signal detection.

### Immunohistochemistry (IHC) assays

All of the 57 tissue samples for the IHC were obtained by needle biopsy or operation after chemotherapy. Formalin-fixed, paraffin-embedded sections were prepared for all tissues. Then sections were deparaffinized in xylene and rehydrated through graded concentrations of alcohol. Next, the slides were treated in 3% H_2_O_2_ in water for 30 min to block endogenous peroxidase activity. Nonspecific staining was blocked by 10% FBS (HyClone, USA) for 1 h. The incubation with the primary anti-ISL1 mouse mAb (5 μg/mL, Developmental Studies Hybridoma Bank, USA) was performed at 4 °C overnight. The next day, tissues were incubated with goat anti-mouse secondary antibody (1:10,000; ZSGB BIO, China) for 2 h at room temperature. We defined two expression levels of the staining: sections with scores of 0–4 were classified as low expression, whereas those with scores of 5–9 were classified as high expression. A positive fluorescence microscope was used to capture images (Olympus BX61, Japan).

### EdU cell proliferation assay

NB cells were transfected with si-NC or si-ISL1 and cultured for 48 h. Then, they were seeded in 12-well plates at a density of 2 × 10^5^ cells per well overnight. The next day, si-NC or si-ISL1 cells were exposed to 50 μM EdU (RiboBio, China) for 2 h at 37 °C. Afterward, cells were stained with 1× Apollo and 1× Hoechst 33342, according to the manufacturer’s instructions (RiboBio, China). Finally, the EdU-stained cells were examined under a confocal microscope (Carl Zeiss, Germany).

### Cell Counting Kit-8 (CCK8) cell proliferation assay

NB cells were seeded in 96-well plates at a density of 4000 cells per well. After incubating for the indicated times, NB cells were then treated with CCK8 (10 µL/well, Apexbio, China) for about 3 h. Then the absorbance at 450 nm was detected by a microplate absorbance reader (Tecan, USA).

### Matrigel invasion assay

Transwell inserts (BD Biosciences, China) coated with 50 μL matrigel per well were prepared to evaluate the invasion ability of NB cells before the experiment. Cells were seeded in 24-well plates with a density of 1 × 10^5^ cells in 200 μL medium without FBS, and were plated into the upper chamber with 8 μm pores. For the lower chamber, a total of 600 μL medium containing 10% FBS was added. After incubation at 37 °C in 5% CO_2_ for about 24 h, non-invaded cells were wiped up with a cotton swab and the invaded cells were fixed with 4% paraformaldehyde for 15 min and then stained with crystal violet for 8 min. Then, they were washed with distilled water and dried in the air. Five fields of each well were randomly selected for imaging, and the number of invaded cells was counted. All images were captured with microscopy (Leica, USA).

### Wound healing assay

A density of 6 × 10^4^ cells/mL of NB cells was seeded in 6-well plates with scratch plug-in components. The cells were cultured with MEM or MEM/F12 medium containing 0.5% FBS at 37 °C in 5% CO_2_ overnight. The next day, the scratch plug-in components were taken out and formed an even wound. Images were captured at 0 and 24 h with a microscope (Leica, USA) at ×20 magnification.

### Apoptosis analysis

Annexin V-FITC Apoptosis Detection Kit (KeyGEN, China) was used for the evaluation of cell apoptosis, following the manufacturer’s instructions. In all, 5 × 10^5^ cells were harvested and were resuspended with 500 μL binding buffer after washing twice with PBS. Then the cells were treated with 5 μL propidium iodide or/and 5 μL Annexin V-FITC for 15 min in the dark. Analysis of the stained cells was performed with flow cytometry (SP6800, Sony, Japan) in 1 h.

### Xenograft tumorigenesis in vivo

NOD/SCID mice at 3–4 weeks of age were used for the experiment in vivo. SK-N-BE (2) cells were inoculated into the inguens of mice (8 × 10^6^ cells per mouse), and tumors could be observable after 2 weeks. Then the mice were randomly divided into two groups: the si-NC group and the si-ISL1 group (with 2ʹ-O me modification). When the volume of the tumor reached about 100 mm^2^ (tumor volume was calculated by the following formula: length × width × width/2), we started to treat the mouse with si-NC or si-ISL1. We injected 2ʹ-O-me-modified si-ISL1 into the tail vein at the dose of 2 mg/kg per animal every 72 h for effective transfection. The si-NC was injected as control. Tumor weight and size were measured every 2 days. All the mice were euthanized 6 weeks after inoculation since one mouse became moribund.

### Statistical methods

SPSS 22.0 software (SPSS, Chicago, IL, USA) was used to analyze the statistical data. The Student’s *t*-test was used to evaluate statistical significance between different groups, *χ*^2^ test was used to assess the relationship between ISL1 expression level and clinicopathological variables. Kaplan-Meier curves were used for survival analysis. OS was defined as the interval between initial diagnosis and the date of death or last follow-up time; while PFS was defined as the time interval from the initial diagnosis to the date of progression or death. *P*-values < 0.05 were considered statistical significance.

## Results

### Identification and functional enrichment of overlapped DEGs

**Fig. 1:**
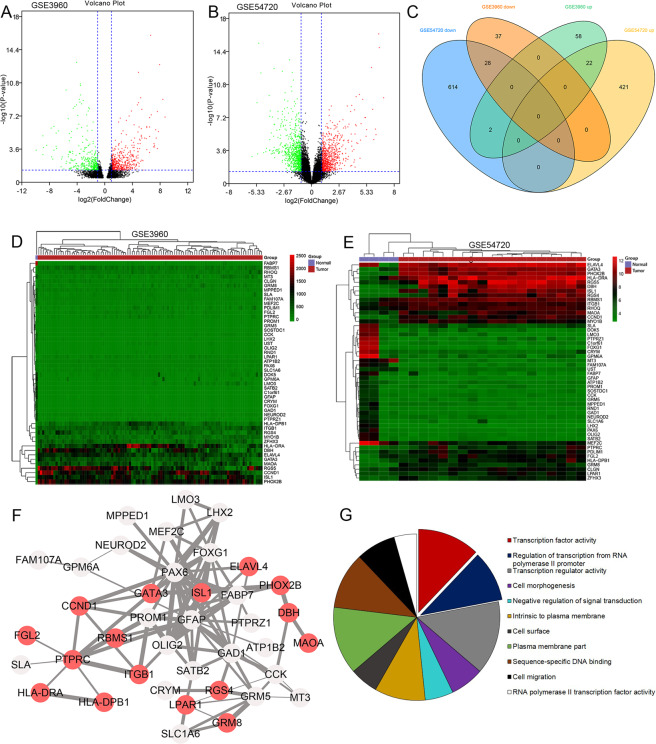
Bioinformatics analysis identified overlapped DEGs between GSE3960 and GSE54720 and performed functional enrichment. **A**, **B** Volcano plots of GSE3960 and GSE54720, where red dots, green dots, and black dots indicate upregulated, downregulated, and not differentially expressed genes, respectively. **C** Venn diagram shows 22 upregulated and 28 downregulated genes. **D**, **E** Heatmaps of common DEGs between NB and normal controls in GSE3960 and GSE54720. **F** PPI network of overlapping DEGs at the protein level. **G** GO analysis reveals 50 overlapping DEGs are mostly enriched in transcription factor activity (*P* = 1.06 × 10^−3^) and regulation of transcription from RNA polymerase II promoter (*P* = 1.55 × 10^−3^). *n* = 50 genes, 41 Gene Ontology classification.

To determine the NB gene expression profiles, we performed bioinformatics mining based on two GEO databases, including GSE3960 and GSE54720. As shown in Fig. 1A, B, we identified 82 and 443 upregulated DEGs, 65 and 644 downregulated DEGs in GSE3960 and GSE54720, respectively. As shown in the Venn diagram (Fig. 1C), a total of 22 upregulated and 28 downregulated genes were found to be significantly differentially expressed between the two datasets. The heatmaps were shown in Fig. 1D, E, each row represents a gene, and each column represents a tissue sample. The color indicates the expression levels of DEGs between NB and normal tissues.

To analyze the signaling pathways involved in relevant DEGs, we performed GO analysis and found that the 50 overlapping DEGs were mainly associated with (were mostly enriched in) transcription factor activity, regulation of transcription from RNA polymerase II promoter, cell morphogenesis, and negative regulation of signal transduction, intrinsic to the plasma membrane, cell surface, and sequence-specific DNA binding (Fig. 1G).

### PPI network construction and hub genes’ identification

**Fig. 2:**
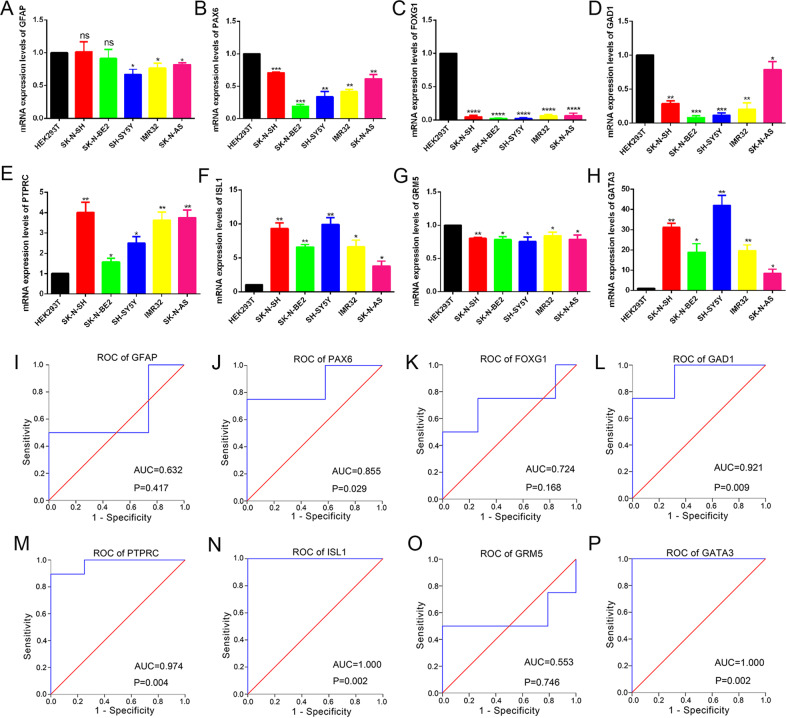
The eight hub genes of identification. **A** The mRNA expression levels of the top eight hub genes were determined in HEK293T cells and 5 NB cell lines by qRT-PCR. **B** Validation of ROC results of the 8 hub genes in NB based on GSE54720 (*P < 0.05, **P < 0.01, ***P < 0.001, ****P < 0.0001).

Based on the STRING database and Cytoscape software, a total of 39 of the 50 commonly altered DEGs were filtered into the PPI network, which contained 39 nodes and 96 edges (Fig. 1F). In PPI, edge widths correspond to the level of confidence in interactions, and the confidence STRING score greater than 0.4 was selected for constructing PPI networks. The top 8 significant nodes, including GFAP, PAX6, FOXG1, GAD1, PTPRC, ISL1, GRM5, and GATA3 were identified as hub genes using the CytoHubba plugin. According to the dataset, all hub genes were downregulated, excluding PTPRC, ISL1, and GATA3. We further used the TCGA TARGET GTEx cohort database to verify expression levels of the top 8 genes. With TCGA TARGET GTEx cohort, we verified the expression of these genes in NB tissues that was significantly different from that in normal tissues, and both ISL1 and GATA3 were significantly higher in NB tissues than in normal tissues (Supplementary Fig. 2). qRT-PCR analysis demonstrated mRNA expression levels of 8 hub genes, in which ISL1 and GATA3 were highly expressed in 5 NB cell lines compared with that in HEK293T (Fig. 2A–H).

Next, the top 8 aberrantly expressed hub genes based on the GSE54720 were sent for ROC analysis. As shown in Fig. 2I–P, these 8 hub genes had high diagnostic values to distinguish NB from normal tissues with area under the ROC curve (AUC) > 0.80, except for GFAP, FOXG1, and GRM5. Besides, the AUC of ISL1 and GATA3 were both 1.000 (*P* = 0.002, Fig. 2N, P). This indicated that ISL1 and GATA3 had promising diagnostic efficiency for NB.

### Overexpression of ISL1 and GATA3 were correlated with poor prognosis in NB patients

As shown in Supplementary Tables 1 and 2, we analyzed the relationships between the expression of ISL1 and GATA3 and clinical–pathological features based on the database. We found that ISL1 and GATA3 expression had a close correlation with histology (*χ*^2^ = 65.164, *P* < 0.01, and *χ*^2^ = 87.274, *P* < 0.01, respectively) (Supplementary Tables 1 and 2). Besides, the expression level of GATA3 was closely related with age (*χ*^2^ = 7.162, *P* = 0.007, Supplementary Table 2) and INSS stage (*χ*^2^ = 8.423, *P* = 0.004, Supplementary Table 2). However, there was no significant correlation between ISL1 and sex, age, N-Myc status, relapse, INSS stage, or COG risk group (*P* > 0.05, Supplementary Table 1). Similarly, GATA3 expression was not correlated with the patient’s sex, N-Myc status, relapse, and COG risk group (*P* > 0.05, Supplementary Table 2).

To verify the prognostic value of ISL1 and GATA3, data from TARGET-NBL, GSE62564, and E-TABM-38 were analyzed. As shown in Supplementary Fig. 1, compared to high ISL1 expression, patients with low ISL1 expression had significantly better EFS and OS in TARGET-NBL (*P* = 0.0472 and *P* = 0.0388, Supplementary Fig. 1A, B) and the same results were found in GSE62564 (*P* = 0. 013 and *P* = 0. 026, Supplementary Fig 1C, D,) and E-TABM-38 (*P* = 0.049 and *P* = 0.017 Supplementary Fig. 1E, F). The prognostic value of GATA3 was shown in Supplementary Fig. 1G, H (*P* = 0.0255 and *P* = 0.0320). Taken together, these findings indicated that high expression of ISL1 and GATA3 were poor prognostic factors.

**Fig. 3:**
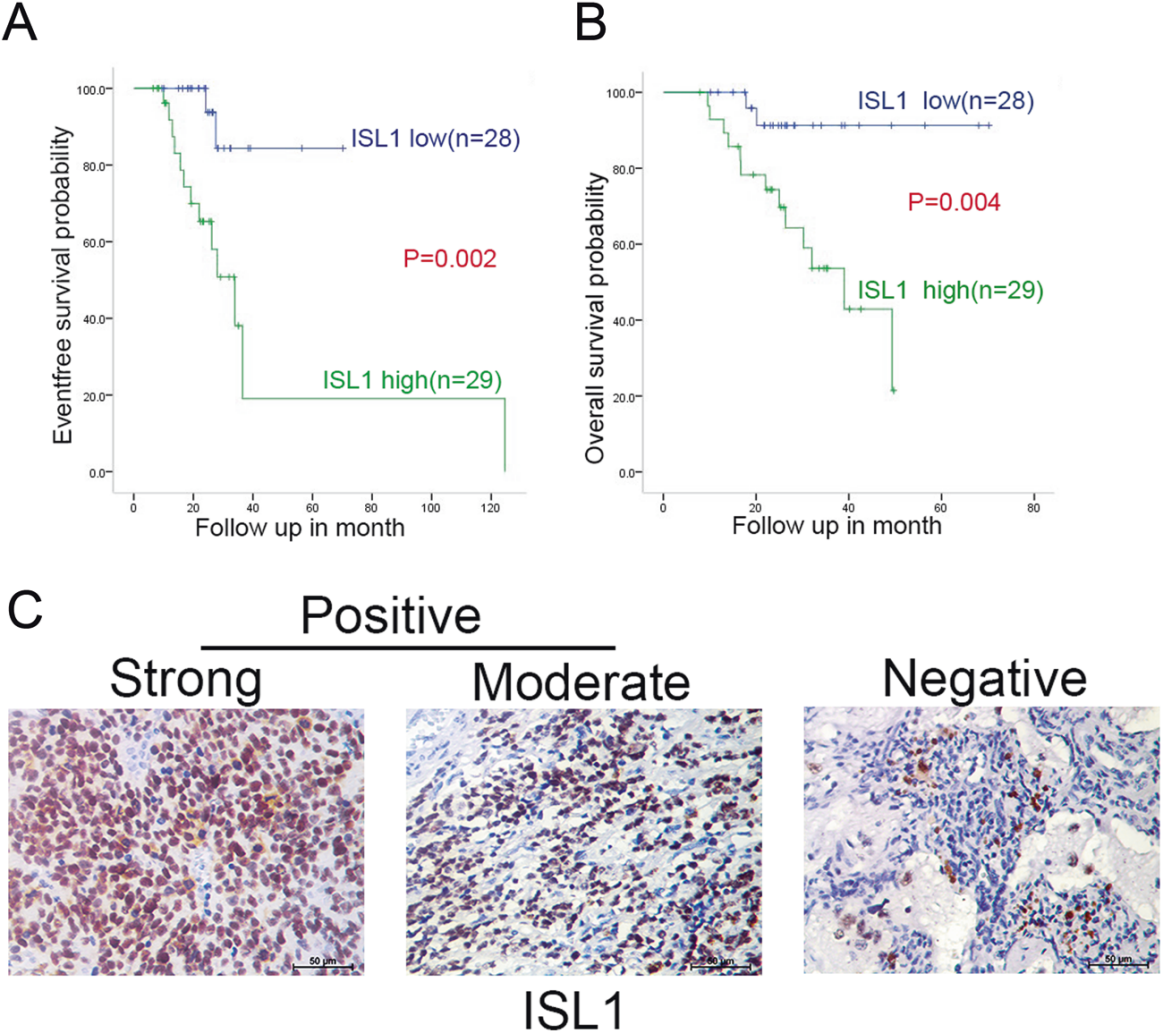
Association of ISL1 expression with the EFS and OS in clinical NB patients. **A**, **B** Kaplan-Meier survival curves about EFS and OS in 57 NB patients expressing different ISL1 levels (***P < 0.001). **C** Typical pictures of IHC analysis in NB tissues with various expression levels of ISL1 (scale bar, 1.0 mm).

As two important transcription factors in NB, functions of ISL1 and GATA3 in NB have not been clearly elucidated yet. Interestingly, here we verified that in si-ISL1-transfected NB cells, GATA3 was downregulated compared with si-NC-transfected NB cells. In turn, we transfected NB cells with si-GATA3, but there seemed no obvious change in ISL1 after transfection. We supposed that ISL1 may be located in the upstream of GATA3 (Supplementary Fig. 3). In view of its important role, we selected ISL1 as the target gene, and the prognostic value of ISL1 was also verified in clinical patients from our department. Detailed characteristics of these 57 NB patients are listed in Table 1. The Kaplan-Meier survival curves showed the prognostic role of ISL1; high expression of ISL1 was a poor prognostic factor of NB (Fig. 3A, B). Typical images of IHC analysis were presented in Fig. 3C, and the para-cancerous tissue was used as a control. The expression level of ISL1 was closely related to COG risk group (*P* < 0.005, Table 1).

**Table 1:** The relationship between *ISL1* expression and clinical–pathological features in 57 NB patients from our department.

Clinical characteristics	Total no. of patients	No. of patients		*χ*2	*P*-value
		Lower (*n* = 28)	Higher (*n* = 29)		
Sex					
Male	32	16	16	0.022	0.88
Female	25	12	13		
Age					
<18 months	12	4	8	1.516	0.218
≥18 months	45	24	21		
Relapse					
No	34	20	14	3.173	0.075
Yes	23	8	15		
INSS stage					
1, 2, 4 s	10	7	3	2.115	0.146
3, 4	47	28	29		
COG risk group					
Low risk	3	2	1	10.493	0.005
Intermediate risk	8	8	0		
High risk	46	18	28		
LDH					
≥245	42	18	24	2.507	0.113
<245	15	10	5

### ISL1 was upregulated in NB cells and it regulated cell proliferation, migration, and apoptosis in vitro

**Fig. 4:**
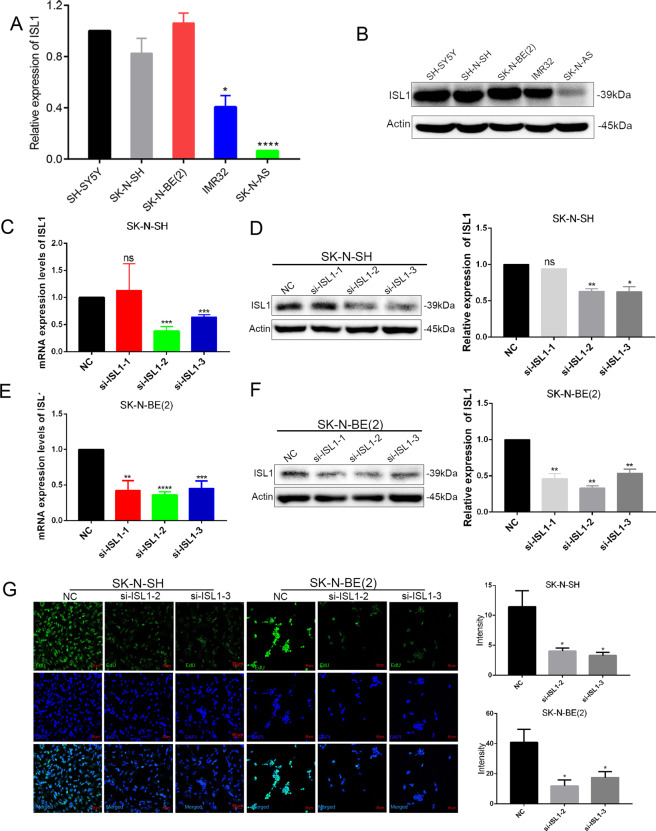
ISL1 was elevated in NB cell lines and regulated NB cell proliferation in vitro. **A** The mRNA expression levels of ISL1 were determined in 5 NB cell lines. **B** The protein expression levels of ISL1 were determined by WB analysis in 5 NB cell lines. **C, D** The transfection efficiency of ISL1 mRNA expression and protein expression in SK-N-SH cells. **E**, **F** The transfection efficiency of ISL1 mRNA expression and protein expression in SK-N-BE (2) cells. **G** The EdU assay in SK-N-SH and SK-N-BE (2) NB cell lines that transfected with si-NC, si-ISL1-2, or si-ISL1-3.

To further verify the expression level of gene ISL1, we performed qRT-PCR and WB analysis, and the SH-SY5Y cell line was used as a positive control. As illustrated in Fig. 4A, B, SK-N-SH and SK-N-BE (2) cell lines have an almost equal or higher expression level of ISL1 as compared with SH-SY5Y; whereas IMR32 or SH-N-AS cells have a relatively lower expression level of ISL1 than SH-SY5Y.

**Fig. 5:**
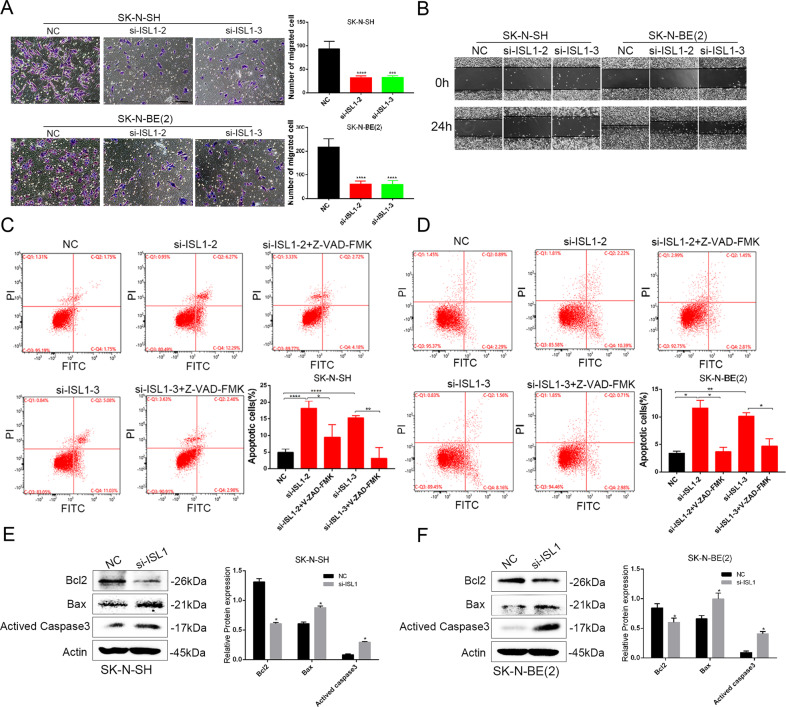
ISL1-regulated NB cell invasion, migration, and apoptosis in vitro. **A** Comparison of the invasion capacity in SK-N-SH and SK-N-BE (2) cell lines (*P* < 0.001). **B** Comparison of the migration capacity by wound healing assay in SK-N-SH and SK-N-BE (2) cell lines (*P* < 0.05). **C**, **D** Apoptosis analysis by flow cytometry in SK-N-SH and SK-N-BE (2) cell lines (*P < 0.05, **P < 0.01, ***P < 0.001), Q2 and Q4 regions represent the rate of early and late apoptotic cells, respectively. **E**, **F** WB analysis of apoptotic-related markers of 2 NB cell lines after knockdown of ISL1 by si-ISL1-2.

Next, to explore the specific function of ISL1 in NB cells, we knockdown ISL1 with si-RNA in SK-N-SH and SK-N-BE (2) cell lines. A total of 3 siRNAs were used for transfection (Fig. 4C–F), and we selected si-ISL1–2 and si-ISL1-3 for further function investigation since these two siRNAs had a better knockdown efficiency. For the cell proliferation study, we performed an EdU test according to instructions, and the results showed that si-ISL1-transfected NB cells had a lower proliferation rate than si-NC-transfected NB cells, based on lower fluorescence intensity and density (Fig. 4G). The migration assays showed a lower invasion ability of si-ISL1-transfected cells than the si-NC-transfected cells (Fig. 5A). Wound healing assays revealed that si-ISL1-transfected cells had a shorter migration distance than si-NC-transfected cells (Fig. 5B).

Apoptosis analysis revealed a slightly higher percentage of apoptotic cells in si-ISL1 NB cells than si-NC cells (Fig. 5C, D, *P* < 0.05). To find the possible mechanism of how that ISL1 regulated apoptosis of NB cells, we treated NB cell lines with 20 μM pan caspase inhibitor Z-VAD-FMK for 48 h, and the results showed that the apoptosis induced by si-ISL1 could be reversed by Z-VAD-FMK. We supposed that ISL1-regulated NB cell apoptosis may be associated with the classic caspase pathway. To further verify our hypothesis, we performed WB analysis and used the si-ISL1-2 for knockdown of ISL1. Results demonstrated that compared to the NC group, the si-ISL1-transfected cell had decreased expression levels of Bcl2, but an increased level of Bax and activated caspase3 between the two groups of NB cells (Fig. 5E, F, *P* < 0.05).

### Downregulation of ISL1 in SK-N-BE (2) cells suppressed tumorigenesis in vivo

**Fig. 6:**
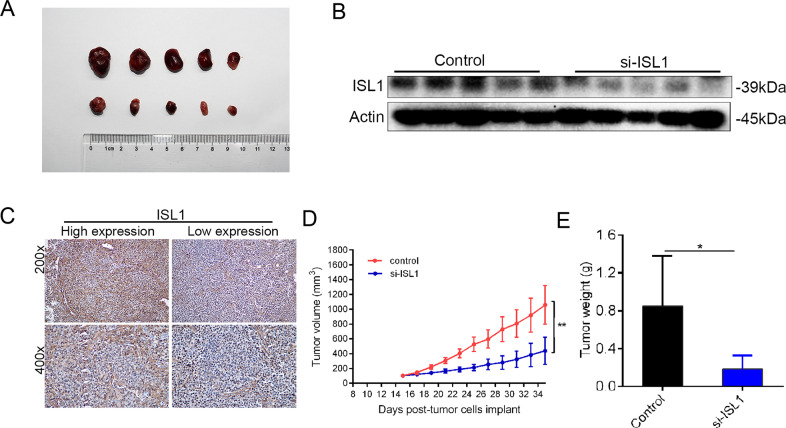
Downregulation of ISL1 in SK-N-BE (2) cells suppressed tumorigenesis in vivo. **A** Tumors from the NOD/SCID mice after subcutaneous injection with the same number of 8.0 × 10^6^ SK-N-BE (2) cells per mouse into the inguen (*n* = 5 per group). All the mice were sacrificed 6 weeks later, and the primary tumor weight (*P* < 0.05) and volume (*P* < 0.01) were evaluated. **B** WB analysis of ISL1 in primary tumors of two groups. **C** IHC analysis of ISL1 in primary tumors of two groups. **D**, **E** Changes in tumor volumes and tumor weight in two groups of mice (*P < 0.05, **P < 0.01).

To explore the function of ISL1 in NB tumorigenesis in vivo, SK-N-BE (2) cells were inoculated into the inguens of NOD/SCID mice. The mice were then randomly divided into two groups: the si-NC group and the si-ISL1 group and were treated with si-NC or si-ISL1 by intravenous injection, respectively. Tumor weight and size were measured every 2nd day. According to Fig. 6A, tumor sizes in the si-ISL1 injection group were smaller than those of the si-NC group. The corresponding expression level of ISL1 in mice is shown in Fig. 6B. Typical pictures of IHC are presented in Fig. 6C. As shown in Fig. 6D, E, our results demonstrate that ISL1 promoted tumorigenesis through increasing tumor weight and volume in vivo, which are in good agreement with the results in vitro.

### ISL1 promoted EMT process via PI3K/AKT signaling pathway in NB

**Fig. 7:**
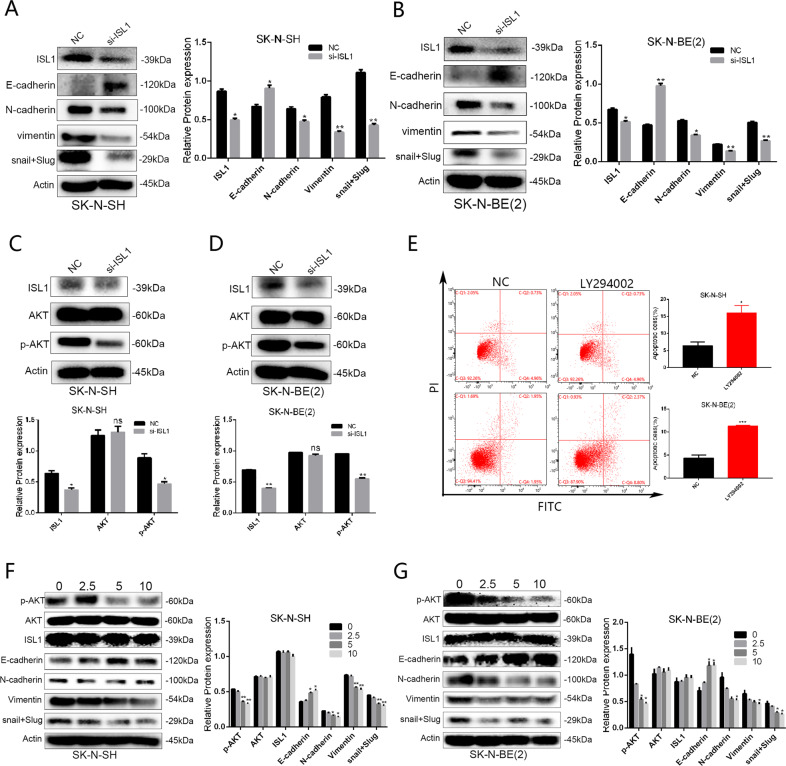
ISL1 promoted EMT in NB cell lines through PI3K/AKT signal pathway. **A**, **B** Expression changes of EMT-associated markers (epithelial marker: E-cadherin, mesenchymal markers: N-cadherin, vimentin, and Slug) in SK-N-SH and SK-N-BE (2) cells after ISL1 was downregulated by si-ISL1-2. **C**, **D** WB analysis of total AKT and p-AKT in SK-N-SH and SK-N-BE (2) cell lines after transfection with si-ISL1-2. **E** Apoptosis analysis of SK-N-SH and SK-N-BE (2) cell lines treated with PI3K inhibitor LY294002 for 48 h. **F** Comparison of ISL1, AKT, p-AKT protein, and EMT-associated markers’ expression in SK-N-SH and SK-N-BE (2) cells with or without using PI3K inhibitor LY294002 (concentration at 0, 2.5, 5, and 10 μM. *P < 0.05, **P < 0.01, ***P < 0.001).

EMT was reported to be closely related with tumor metastasis, and it confers on NB cells invasive phenotype with increased tumor-initiating and metastatic potential [21, 22]. As an important transcription factor for differentiation of NB cells, we wondered whether ISL1 regulated EMT in NB, and we detected the expression of EMT markers (E-cadherin, N-cadherin, snail + Slug, and vimentin) by WB analysis. The results demonstrated that the protein levels of epithelial marker E-cadherin increased while the mesenchymal markers, such as N-cadherin, vimentin, and snail + Slug, decreased in si-ISL1 NB cells compared with si-NC NB cells (Fig. 7A, B). These results indicated that ISL1 may promote EMT in NB cells.

The activated PI3K/AKT signaling pathway is considered to play a significant role in the development and regulation of many tumors; while EMT endows cells with the ability to metastasize and invade. By the mediation of several transcriptional and growth factors, the activated PI3K/AKT signaling pathway was reported to play a significant role in EMT regulation. Here we assumed that ISL1 promoted EMT in NB may be via the PI3K/AKT signaling pathway.

To confirm this hypothesis, we performed WB analysis, and our results showed that compared with si-NC NB cells, si-ISL1 NB cells had lower phosphorylated AKT (p-AKT) expression level; while expression level of AKT had no significant difference between these two groups (Fig. 7C, D).

Next, we used the PI3K inhibitor LY294002 in NB cells for about 48 h. We found that NB cells treated with LY294002 had a higher apoptosis rate than cells without LY294002 (Fig. 7E). In other words, PI3K inhibitor LY294002 could alone induce apoptosis of NB cells. This result indicated that PI3K/AKT signaling pathway may at least partly be regulated by ISL1 during the tumorigenesis process in NB. Results of WB showed that after treated with LY294002, protein levels of epithelial marker E-cadherin increased while the mesenchymal markers decreased in a dose-dependent way (Fig. 7F, G). Based on the above results, we concluded that ISL1 promoted EMT in NB via activating PI3K/AKT signaling pathway.

### Activation of PI3K/AKT pathway by ISL1 was depended on AURKA

**Fig. 8:**
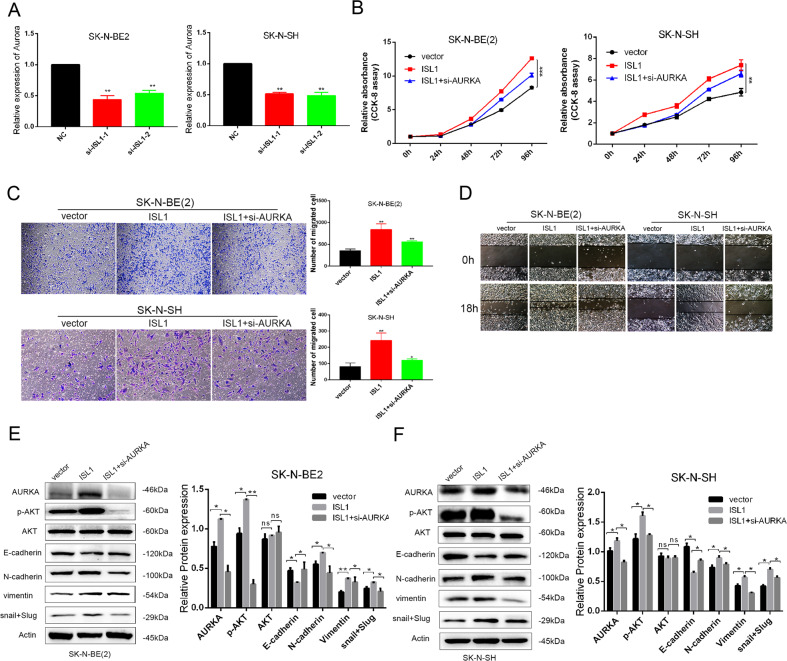
Activation of PI3K/AKT pathway by ISL1 was depended on AURKA. **A** mRNA expression level of AURKA after knockdown of ISL1 in SK-N-BE (2) and SK-N-SH cells. **B** CCK8 proliferation assay of ISL-overexpressed-SK-N-BE (2) and ISL-overexpressed-SK-N-SH after knockdown of AURKA with si-AURKA. **C** Comparison of the invasion capacity in ISL-overexpressed-SK-N-BE (2) and ISL-overexpressed-SK-N-SH after knockdown of AURKA with or without si-AURKA. **D** Comparison of the migration capacity in ISL-overexpressed-SK-N-BE (2) and ISL-overexpressed-SK-N-SH after knockdown of AURKA with or without si-AURKA. **E**, **F** Comparison of ISL1, AKT, p-AKT protein, and EMT-associated markers expression in ISL-overexpressed-SK-N-BE (2) and ISL-overexpressed-SK-N-SH after knockdown of AURKA with or without si-AURKA (*P < 0.05, **P < 0.01, ***P < 0.001).

We found that AURKA was downregulated in si-ISL1-transfected NB cells (Fig. 8A). AURKA is a serine-threonine kinase known to phosphorylate AKT and plays an important role in the survival of NB. We wondered if the activation of AKT by ISL1 was depended on AURKA. To verify this hypothesis, we established ISL1-overexpressed NB cells by lentivirus transduction, and then blocked AURKA by knockdown AURKA gene with a siRNA. We demonstrated that ISL1 overexpressing significantly enhanced cell proliferation (Fig. 8B) and migration (Fig. 8C, D), increased AKT phosphorylation and EMT transformation (Fig. 8E, F). While AURKA knockdown attenuated the function of ISL1 overexpression in the regulation of cell proliferation, AKT phosphorylation, and EMT (Fig. 8E, F).

## Discussion

Large-scale genomic studies have revealed that there are few frequent mutations in NB. The major mutations only include ALK (9. 2%), PTPN11 (2. 9%), ATRX (2. 5%), MYCN (1. 7%), and NRAS (0.83%) [33]. The lack of frequent mutations challenges current therapeutic strategies that rely on targets with high mutation frequency and makes many researchers turning their interest into abnormal transcriptome of NB. For example, studies have found that members of the core transcription factor loop promoted by super-enhancers, including PHOX2B, HAND2, GATA3, TBX2, ISL1, YAP1, and AP1, are abnormally overexpressed in NB [34, 35]. Using bioinformatics analysis, we determined the top 8 differentially expressed genes (DEGs) in NB, including GFAP, PAX6, FOXG1, GAD1, PTPRC, ISL1, GRM5, and GATA3. Among them, our analysis showed that ISL1 and GATA3 are poor prognostic factors of NB. Previous studies showed ISL1 can physically interact with GATA3, and they synergistically regulate several crucial oncogenic pathways in NB [36]. In the present study, we demonstrated that ISL1 is located at upstream and regulates the expression of GATA3. As a result, we preferred ISL1 as the research object prior to GATA3. However, we believe that the study of the function of either ISL1 or GATA3 in NB is of great significance.

ISL1, also known as insulin gene enhancer binding protein 1, was first discovered in rat insulinoma cell line by Karlsson in 1990 and sequenced [37]. ISL1 is a member of LIM domain protein family, which contains three highly conservative domains: two tandem LIM domains and one homologous domain, which mediate the interaction between protein and protein, and protein and DNA, respectively [38]. ISL1 is an important transcription factor that plays an essential role in the embryonic development of heart and sympathetic nervous system, and influences various cell differentiations [36, 39, 40]. Compared with normal tissues, ISL1 is significantly upregulated in various cancers. ISL1 is involved in caner progression and is considered as a prognostic factor of multiple cancers [11–18]. In this study, we identified ISL1 as a novel oncogene acting through activation of PI3K/AKT signaling pathway in NB. In the experiment we observed that, the proportion of apoptotic cells was much less than the extent of cell number reduction. This indicated that ISL1 knockdown caused a decrease in the number of cells by reducing the proliferation ability and inducing apoptosis of NB cells together. The apoptosis induced by ISL1 knockdown was in a caspase-dependent manner and could be prevented with caspase inhibitor Z-VAD-FMK. We also showed that PI3K inhibitor alone can induce apoptosis in NB, which is consistent with previous reports [41]. This helps to explain that ISL1 promoted tumorigenesis and induced apoptosis by activation of PI3K/AKT signaling pathway.

AURKA is a widespread protein kinase that plays key roles during cell division and is considered as a potent oncogene and target for cancer therapy [42]. It is overexpressed in NB and indicates a poor outcome [43]. The clinical studies of AURKA inhibitors in patients with refractory NB are in progress [44]. Many studies have shown that AURKA can phosphorylate AKT [45] and promote EMT transition of cancers [46, 47]. In the present study, we showed that cell proliferation, phosphorylation of AKT, and EMT transition induced by ISL1 overexpression all could be attenuated by AURKA knockdown in both MYCN-amplification cells SK-N-BE (2) and MYCN-nonamplification cells SK-N-SH. Thus, we revealed that ISL1 promotes tumorigenesis of NB in an AURKA-dependent manner regardless of MYCN status. In addition, AURKA is known to locate in the microtubule of centrosome and spindle during cell division, which ensures normal mitosis. Whether ISL1 also plays a role in the mitosis of cancer cells needs further study.

ISL1 directly or indirectly regulates many genes essential for the proliferation and differentiation of sympathetic neurons, many of them are related to the pathogenesis of NB [39]. It acts on the upstream of several oncogenes and pathways, including LMO1 and LIN28B, which is crucial for the proliferation and differentiation of NB [36]. Interestingly, although metastatic NB cells SK-N-BE (2) (MYCN amplification) and SK-N-SH (MYCN nonamplification) express ISL1 themselves, when we increase expression of ISL1 by the overexpression vector, the ability of cell proliferation and migration is further enhanced. These results confirm that ISL1 is an oncogene of NB. Therefore, we believe that further study on the upstream and downstream of ISL1, such as epigenetic regulatory factors, gene reprogramming process of proliferation and differentiation, or cooperation with other known oncogenes, etc. is helpful to better understand the pathogenesis of NB and find novel therapeutic targets.

## Supplementary information

Supplemental Fig. 1.

Supplemental Fig. 2.

Supplemental Fig. 3.

Supplementary Table 1.

Supplementary Table 2.

Supplementary Table 3.

## Data Availability

The authenticity of this article has been validated by uploading the key raw data to the Research Data Deposit (RDD) public platform (www.researchdata.org.cn), with the approval RDD number as RDDB2021001098.
