# Supplementary figures and images for: ISL1 promoted tumorigenesis and EMT via Aurora kinase A-induced activation of PI3K/AKT signaling pathway in neuroblastoma
Source: Cell Death Dis. 2021 Jun 15;12(6):620. doi: 10.1038/s41419-021-03894-3 (PMC8206128; doi:10.1038/s41419-021-03894-3)

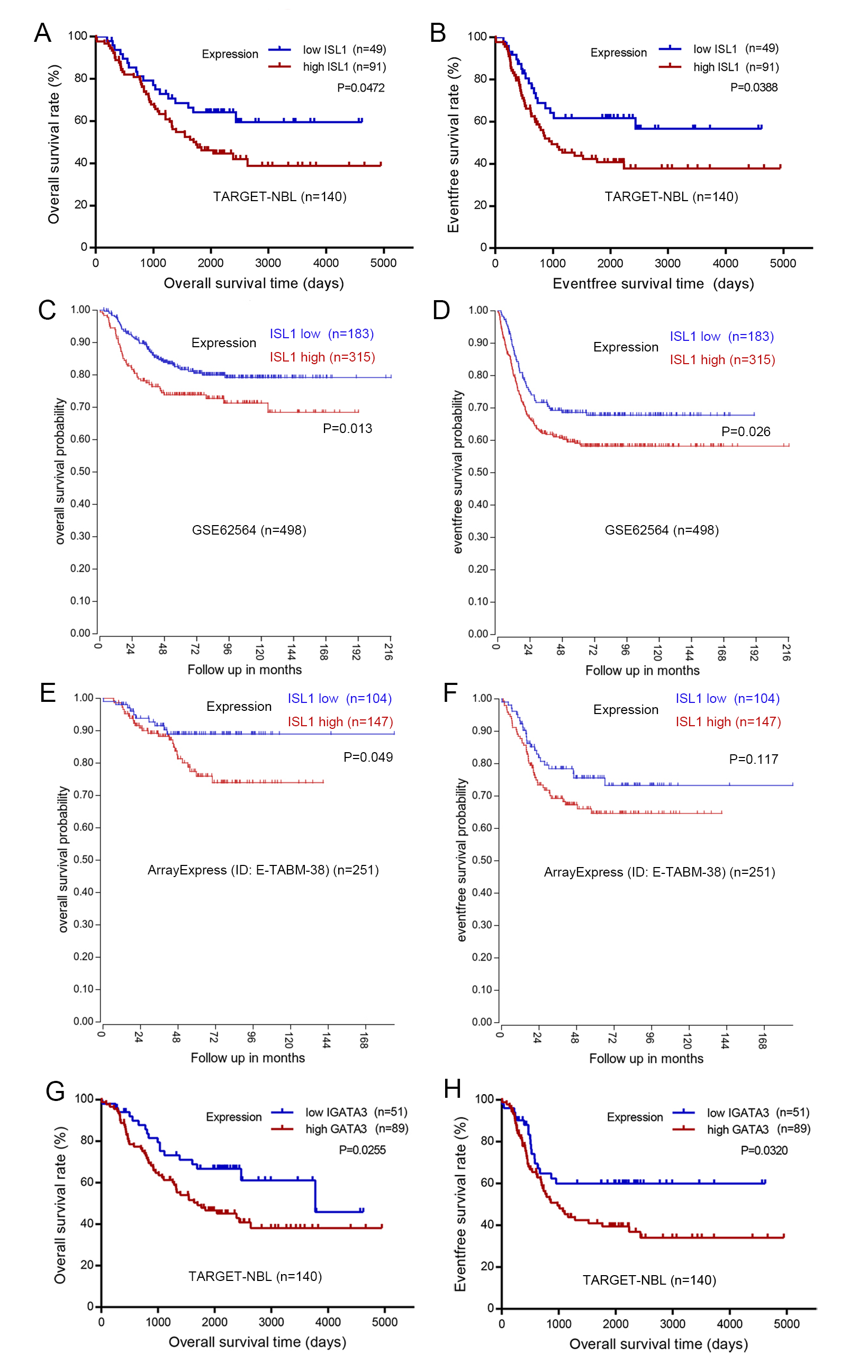

Supplement: Supplementary file 1 — Supplemental Fig. 1. [file 41419_2021_3894_MOESM1_ESM.tif]

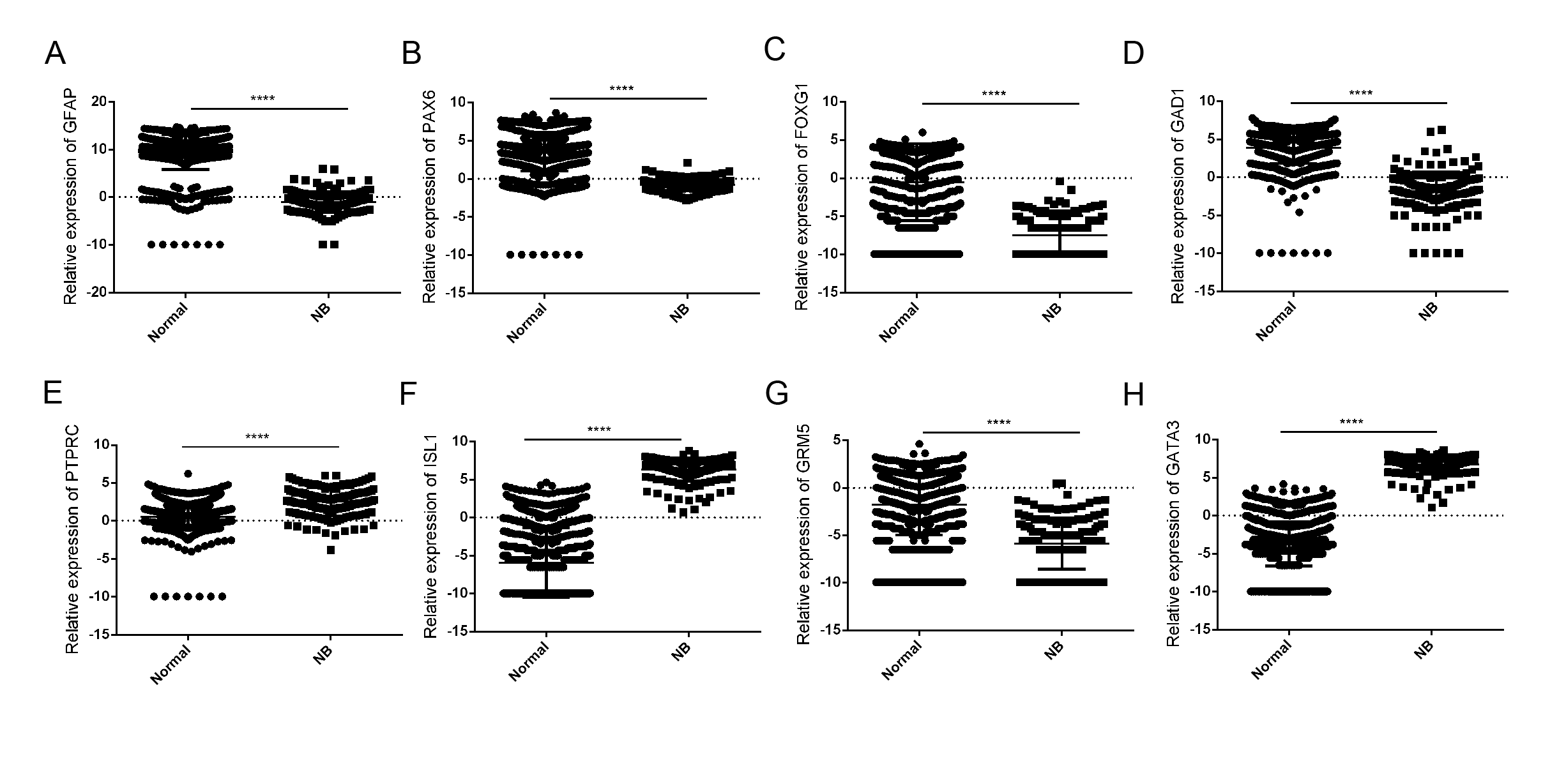

Supplement: Supplementary file 2 — Supplemental Fig. 2. [file 41419_2021_3894_MOESM2_ESM.tif]

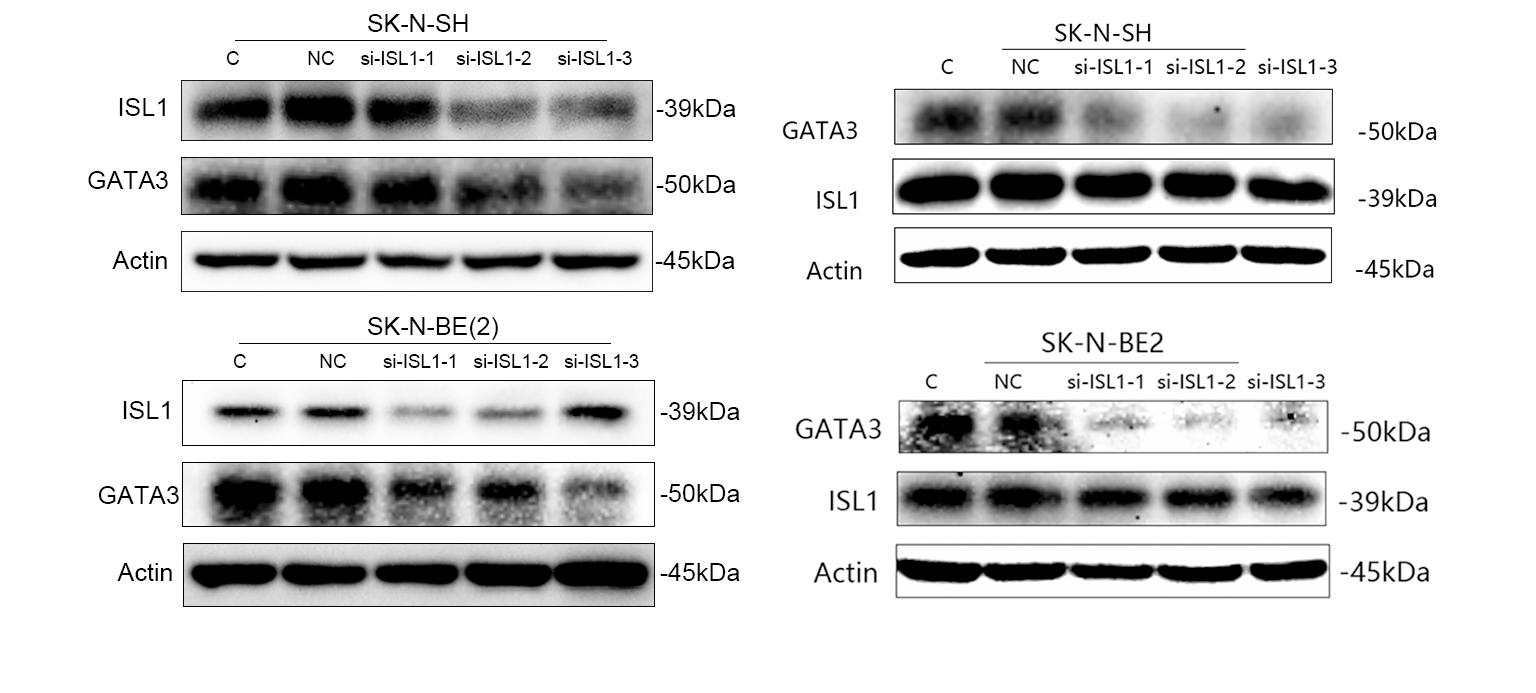

Supplement: Supplementary file 3 — Supplemental Fig. 3. [file 41419_2021_3894_MOESM3_ESM.tif]
