# Supplementary material for: ISL1 promoted tumorigenesis and EMT via Aurora kinase A-induced activation of PI3K/AKT signaling pathway in neuroblastoma
Source: Cell Death Dis. 2021 Jun 15;12(6):620. doi: 10.1038/s41419-021-03894-3 (PMC8206128; doi:10.1038/s41419-021-03894-3)
Supplement: Supplementary file 4 — Supplementary Table 1. [file 41419_2021_3894_MOESM4_ESM.docx]

**Supplementary Table1.** The relationship between *ISL1* expression and clinical pathological features in 140 neuroblastoma patients from TARGET-NBL.

| Clinical  Characteristics | NO. of patients | NO. of patients | | χ2 | P-value |
| --- | --- | --- | --- | --- | --- |
|  |  | Lower (n=49) | Higher (n=91) |  |  |
| **Sex** |  |  |  |  |  |
| Male | 83 | 29 | 54 | 0 | 0.986 |
| Female | 57 | 20 | 37 |  |  |
| **Age** |  |  |  |  |  |
| <18 months | 24 | 9 | 15 | 0.08 | 0.778 |
| ≥18 months | 116 | 40 | 76 |  |  |
| **N-myc status** |  |  |  |  |  |
| Unamplification | 111 | 40 | 71 | 0.253 | 0.615 |
| Amplification | 29 | 9 | 20 |  |  |
| **Relapse** |  |  |  |  |  |
| No | 82 | 28 | 54 | 0.063 | 0.801 |
| Yes | 58 | 21 | 37 |  |  |
| **INSS stage** |  |  |  |  |  |
| 1, 2, 4s | 22 | 9 | 13 | 0.401 | 0.527 |
| 3, 4 | 118 | 40 | 78 |  |  |
| **COG Risk Group** |  |  |  |  |  |
| Low Risk | 14 | 5 | 9 | 1.891 | 0.388 |
| Intermediate Risk | 8 | 1 | 7 |  |  |
| High Risk | 118 | 43 | 75 |  |  |
| **Histology** |  |  |  |  |  |
| Favorable | 23 | 11 | 12 | 65.164 | ＜0.01 |
| Unfavorable | 108 | 37 | 71 |  |  |
| Unknown | 9 | 1 | 8 |  |  |
