# Supplementary material for: ISL1 promoted tumorigenesis and EMT via Aurora kinase A-induced activation of PI3K/AKT signaling pathway in neuroblastoma
Source: Cell Death Dis. 2021 Jun 15;12(6):620. doi: 10.1038/s41419-021-03894-3 (PMC8206128; doi:10.1038/s41419-021-03894-3)
Supplement: Supplementary file 5 — Supplementary Table 2. [file 41419_2021_3894_MOESM5_ESM.docx]

**Supplementary Table2**. The relationship between *GATA3* expression and clinical pathological features in 140 neuroblastoma patients from TARGET-NBL.

| Clinical  Characteristics | NO. of patients | NO. of patients | | χ2 | P-value |
| --- | --- | --- | --- | --- | --- |
|  |  | Lower (n=51) | Higher (n=89) |  |  |
| **Sex** |  |  |  |  |  |
| Male | 83 | 28 | 55 | 0.639 | 0.424 |
| Female | 57 | 23 | 34 |  |  |
| **Age** |  |  |  |  |  |
| <18 months | 24 | 3 | 21 | 7.162 | 0.007 |
| ≥18 months | 116 | 48 | 68 |  |  |
| **N-myc status** |  |  |  |  |  |
| Unamplification | 111 | 41 | 70 | 0.06 | 0.807 |
| Amplification | 29 | 10 | 19 |  |  |
| **Relapse** |  |  |  |  |  |
| No | 82 | 31 | 51 | 0.162 | 0.687 |
| Yes | 58 | 20 | 38 |  |  |
| **INSS stage** |  |  |  |  |  |
| 1, 2, 4s | 22 | 2 | 20 | 8.423 | 0.004 |
| 3, 4 | 118 | 49 | 69 |  |  |
| **COG Risk Group** |  |  |  |  |  |
| Low Risk | 14 | 2 | 12 | 5.35 | 0.069 |
| Intermediate Risk | 8 | 5 | 3 |  |  |
| High Risk | 118 | 44 | 74 |  |  |
| **Histology** |  |  |  |  |  |
| Favorable | 23 | 5 | 18 | 87.274 | ＜0.01 |
| Unfavorable | 108 | 41 | 67 |  |  |
| Unknown | 9 | 5 | 4 |  |  |
|  |  |  |  |  |  |
