# Supplementary material for: ISL1 promoted tumorigenesis and EMT via Aurora kinase A-induced activation of PI3K/AKT signaling pathway in neuroblastoma
Source: Cell Death Dis. 2021 Jun 15;12(6):620. doi: 10.1038/s41419-021-03894-3 (PMC8206128; doi:10.1038/s41419-021-03894-3)
Supplement: Supplementary file 6 — Supplementary Table 3. [file 41419_2021_3894_MOESM6_ESM.docx]

**Supplementary Table3.** Primer sequences of qRT-PCR.

| cDNA | Forward primer (5′–3′) | Reverse primer (5′–3′) |
| --- | --- | --- |
| *GFAP* | GCTGCGGCTCGATCAACTCAC | GGTGGCTTCATCTGCTTCCTGTC |
| *PAX6* | CAGAGAAGACAGGCCAGCAACAC | CCAACATGGAGCCAGATGTGAAGG |
| *FOXG1* | CTCACGCTCAACGGCATCTACG | GACAGATTGTGGCGGATGGAGTTC |
| *GAD1* | CATCTTCGTCCGCAACCTCCTC | CGACTCTTCTCTTCCAGGCTGTTG |
| *PTPRC* | CTGCAAGCTGAGGAGCAAGGAAG | CCACCAACTGAAGGCTGAACTGTC |
| ISL1 | TGATGAAGCAACTCCAGCAGCAG | AGCCACCGTCGTGTCTCTCTG |
| *GRM5* | CCTGCTGGCATTCGGCTGTG | GGAGCGGAAGGAAGAGGAGGAG |
| *GATA3* | CACCACCTACCCGCCCTACG | GTTCACACACTCCCTGCCTTCTG |
